# Identification of New Rabies Virus Variant in Mexican Immigrant

**DOI:** 10.3201/eid1412.080671

**Published:** 2008-12

**Authors:** Andres Velasco-Villa, Sharon L. Messenger, Lillian A. Orciari, Michael Niezgoda, Jesse D. Blanton, Chris Fukagawa, Charles E. Rupprecht

**Affiliations:** Centers for Disease Control and Prevention, Atlanta, Georgia, USA (A. Velasco-Villa, L.A. Orciari, M. Niezgoda, J.D. Blanton, C.E. Rupprecht); California Department of Public Health, Richmond, California, USA (S.L. Messenger); Santa Barbara County Public Health Department, Santa Barbara, California, USA (C. Fukagawa)

**Keywords:** rabies, rabies virus, lyssavirus, encephalitis, phylogenetics, emerging viral diseases, border surveillance, dispatch

## Abstract

A novel rabies virus was identified after death in a man who had immigrated from Oaxaca, Mexico, to California, USA. Despite the patient’s history of exposure to domestic and wild carnivores, molecular and phylogenetic characterizations suggested that the virus originated from insectivorous bats. Enhanced surveillance is needed to elucidate likely reservoirs.

Rabies is an acute, progressive, fatal encephalitis caused by viruses in the family *Rhabdoviridae*, genus *Lyssavirus*. Globally, 11 major genotypes have been identified as etiologic agents of this zoonosis (*1*). *Rabies virus* (RV), the type species, is the most widespread and epidemiologically important member of the genus and the only taxon documented in the New World. Major mammalian reservoirs reside in the orders Carnivora and Chiroptera. Several specific RV variants have been characterized from different mammalian hosts, such as dogs, foxes, mongooses, and other carnivores, and bats. Within North America, distinct RV variants have been associated with rabid wildlife, including foxes, coyotes, raccoons, skunks, and multiple species of frugivorous, insectivorous, and hematophagous bats. Antigenic and genetic characterization of RV isolates, combined with traditional epidemiologic methods, is used to infer transmission events when a history of animal exposure is lacking or inconclusive.

In March 2008, a man who had recently immigrated from Mexico went to a hospital in Santa Barbara County, California, USA, where he died. Rabies was suspected, and a history was obtained of prior dog exposure and a confirmed fox bite in Oaxaca, Mexico, 110 days before the onset of neurologic symptoms (*2*). The primary objective of this study was to identify and molecularly characterize the isolate obtained from this patient. Our aims were to determine likely transmission event(s) associated with the case and to demonstrate the need for a better understanding of the biodiversity and epidemiology of RV variants and their reservoirs in this region.

## The Study

**Figure 1:**
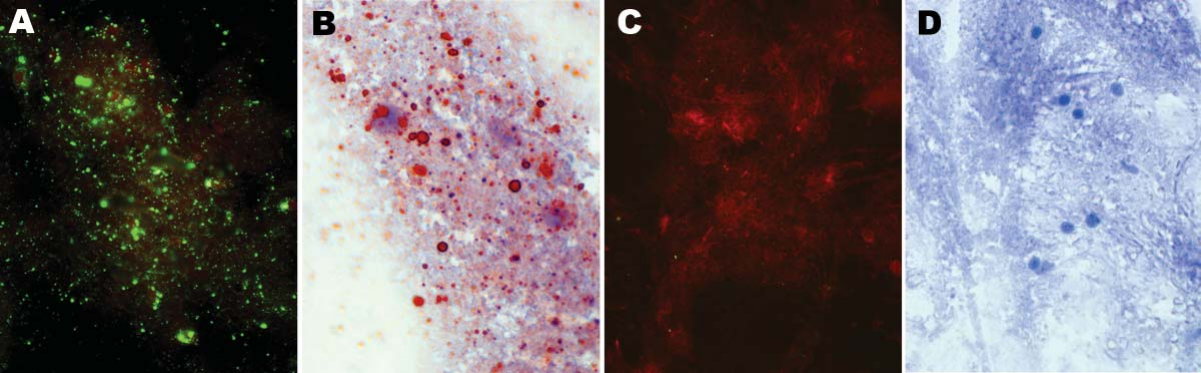
Detection of rabies virus antigen in brain impressions of the patient by direct fluorescent antibody test counterstained with Evans blue, 200× total magnification: A) positive control; B) negative control. Direct rapid immunohistochemistry test counterstained with hematoxylin, 400× total magnification: C) positive control; D) negative control.

At autopsy, brain samples were obtained from the patient. Presence of RV antigen on brain tissue was confirmed by the direct fluorescent antibody test (www.cdc.gov/ncidod/dvrd/rabies/Professional/publications/DFA_diagnosis/DFA_protocol-b.htm) and direct rapid immunohistochemical test (*3*) (Figure 1). Antigenic typing was performed with a panel of anti-RV nucleoprotein (N) monoclonal antibodies as described (*4*–*6*). Comparison of the human sample with established reaction patterns of RV variants showed that the closest antigenic match was among insectivorous bat patterns, specifically a unique RV variant in Colorado *Myotis* sp. and several *Tadarida brasiliensis* variants (Appendix Table). Patterns obtained from hematophagous bat-, canine-, and terrestrial carnivore–associated RV variants were not consistent with the pattern obtained from the patient.

**Figure 2:**
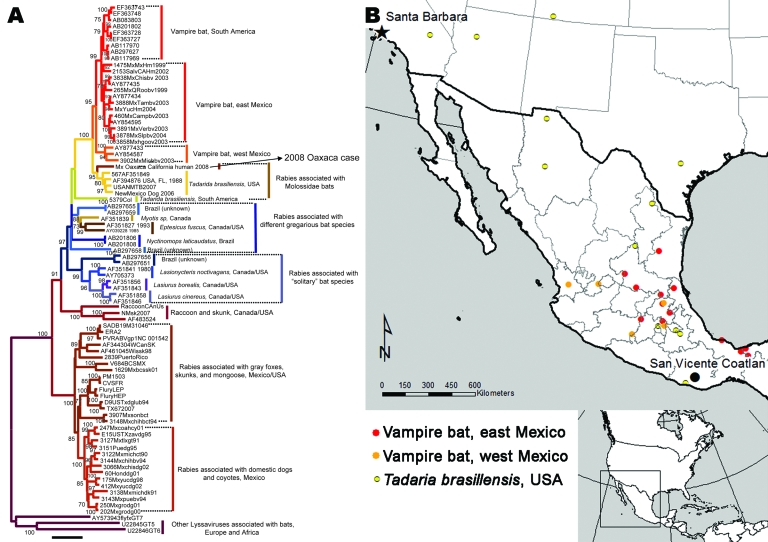
Phylogenetic tree of complete lyssavirus nucleoprotein genes, comparing the patient isolate with representative rabies virus variants associated with common New World animal reservoirs. The map shows the locations of representative samples associated with rabies transmitted by *Tadarida brasiliensis* and vampire bats used in the analysis.

Total RNA was extracted from infected tissue, and the entire N gene was amplified by reverse transcription–PCR in 2 overlapping amplicons, as described (*7*). Phylogenetic analyses were conducted by comparing full and partial RV N sequences with sequences derived from major extant rabies enzootics in both dogs and vampire bats in Mexico, as well as sequences associated with RVs maintained by other bat species and wild terrestrial carnivores from the United States and the Americas (*8*–*10*) (Figure 2). MEGA and BioEdit software were used to perform the phylogenetic reconstructions and sequence analyses (*11*,*12*).

Rabid dogs and vampire bats are the most common sources of exposure for humans in Mexico (www.salud.gob.mx/unidades/cdi/documentos/rabia.pdf). Residual canine rabies enzootics persist in central and southeastern Mexico, whereas vampire bat rabies is found throughout a wide geographic focus, particularly in the tropical and subtropical areas (*8*,*10*). In addition, at least 20 different lineages of RV that are associated with at least 9 bat species have been described in Mexico (*8*). Nevertheless, phylogenetic analyses of the RV obtained from the brain sample of the patient did not support a close relationship with any of the RV variants previously described. The isolate was found to be most closely related with those from Mexican free-tailed bats (*T. brasiliensis*); overall average identity was 95% but clearly segregated in an independent lineage (Figure 2). Given that the average percentage of genetic divergence among previously sequenced members of the *T. brasiliensis* RV clade ranges between 2.4% for the full N over a 20-year period and 1% for the partial N over a 40-year period, the extent of genetic divergence (5%) between the Oaxaca sample and the *T. brasiliensis* RV clade suggests that this isolate represents a new RV variant. Additionally, although the Oaxaca sample shares a distinctive molecular signature with the *T. brasiliensis* RV clade (i.e., conserved amino acid sequence alanine, aspartic acid, and threonine located at positions 377–379 within the N gene), the histidine at position 321, which is unique and highly conserved in members of the *T. brasiliensis* RV lineage, was changed to glutamine in the Oaxaca patient.

Although this patient’s history indicated that he had been bitten near his home (San Vicente Coatlan, district of Ejutla, Oaxaca) by a *costoche* (*2*) (gray fox, *Urocyon cinereoargenteus*), the genetic and phylogenetic analyses did not support a close relationship to any known RV associated with terrestrial carnivores. RVs of major rabies epizootics associated with dogs and other terrestrial carnivores in Mexico and the United States are genetically distinct (average genetic distance 14%–16%) from those in bats throughout North America. Also, although the RV associated with the human case was nested within the monophyletic assemblage of bat RV variants, RV variants phylogenetically closest to this case were still genetically distant. RV variants associated with North American *Tadarida* and vampire bat rabies in Mexico were from 5% to 7% divergent from that of the human case.

Results of partial RV N gene sequence analyses indicated that at least 2 other human rabies cases—one in California in 1995 and the other in Nuevo Leon, Mexico, in 1999—were associated with the *T. brasiliensis* variant. These cases segregated within the monophyletic assemblage that includes enzootic rabies in *T. brasiliensis* bats collected over a period of ≈40 years in the United States and Mexico; the Oaxaca human case sample fell outside the *T. brasiliensis* clade, forming an independent lineage that was statistically supported in both the partial and full N phylogenetic reconstructions. These results plus the amino acid change found at position 321 in the RV associated with this case reinforced the concept of a new RV lineage associated with an unknown animal reservoir.

## Conclusions

In 2008, a Mexican immigrant with a history of fox bite (*2*) died in California of infection with an RV variant most closely associated with RVs associated with insectivorous bats (*T. brasiliensis*). Both the molecular and phylogenetic characterizations of this RV suggest that this is a new lineage. Although the primary reservoir or most likely origin of this RV was determined to be an insectivorous bat (unknown species), the history of carnivore exposure suggests that a secondary transmitter (vector) could have been involved in the transmission chain, as has been reported in other cases (*13*). One cannot, however, rule out the possibility that the unknown reservoir species of this new RV lineage is, in fact, a different bat species (which could have been involved in the primary transmission after an unnoticed or cryptic exposure) or a terrestrial carnivore (e.g., the biting fox). The establishment of an insectivorous bat-derived RV variant in a terrestrial reservoir (i.e., striped skunk) in northern Arizona has been described (*14*). Enhanced epidemiologic surveillance and intensified research to characterize RV variants and their reservoirs in the region are needed to resolve this intriguing discovery.

## Supplementary Material

Appendix Table Comparative monoclonal antibody (MAb) reactivity of rabies viruses from the patient and likely reservoir host species*
