# Supplementary material for: Identification of New Rabies Virus Variant in Mexican Immigrant
Source: Emerg Infect Dis. 2008 Dec;14(12):1906–8. doi: 10.3201/eid1412.080671 (PMC2634630; doi:10.3201/eid1412.080671)
Supplement: Appendix Table — Comparative monoclonal antibody (MAb) reactivity of rabies viruses from the patient and likely reservoir host species* [file 08-0671_appT-s1.pdf]

Appendix Table. Comparative monoclonal antibody (MAb) reactivity of rabies viruses from the patient and likely reservoir host species*

RV variants†	Rab-N-MAbs																				
	C1	C2	C3	C4	C5	C6	C7	C8	C9	C10	C11	C12	C13	C15	C16	C17	C18	C19	C20	P41	TUA	CR54
Mexico dog	P	P	P	P	P	P	P	P	V	P	P	P	P	P	P	P	N	P	P	P	P	N
Arctic fox	N	P	P	P	P	P	P	P	P	P	P	P	P	N	N	P	N	N	P	P	P	N
Texas fox	P	P	P	P	P	P	P	P	P	P	P	P	P	P	P	P	N	P	P	N	P	N
Arizona fox	P	P	D	P	P	P	P	D	P	N	P	P	P	P	P	D	N	P	P	N	P	N
Eastern raccoon	N	P	P	P	P	P	P	P	P	P	P	V	P	P	P	P	P	P	P	N	P	P
South Central skunk	N	N	P	P	P	P	N	P	P	P	N	N	P	P	P	P	P	P	P	N	N	D
North Central skunk	P	P	P	P	P	P	P	P	P	P	P	P	P	P	P	P	N	P	P	N	P	N
California skunk	P	P	P	P	P	P	P	P	P	P	P	P	P	P	P	P	N	P	P	N	P	N
Eptesicus fuscus I	N	P	P	P	P	P	P	P	P	P	P	P	P	N	P	P	N	N	P	N	P	N
E. fuscus II	N	P	N	P	P	P	P	N	P	N	P	P	V	N	P	N	N	N	P	N	P	N
E. fuscus III	V	P	N	P	P	P	P	N	P	N	P	P	V	V	V	N	V	V	P	N	P	N
Lasiurus borealis	D	P	P	P	P	P	P	P	P	P	P	D	P	N	D	P	N	N	P	N	N	N
L. cinereus	D	P	P	P	P	P	P	P	P	P	P	P	P	N	D	P	N	N	P	N	N	N
Lasionycteris noctivagans	D	P	P	P	P	P	P	P	P	P	P	P	N	N	D	P	N	N	P	N	N	N
Desmodus rotundus	N	P	P	P	P	P	P	P	V	P	P	V	P	V	N	P	N	V‡	P	N	P	N
Tadarida brasiliensis	V	P	P	P	P	P	P	P	P	P	P	P	P	N	N	P	N	N	P	N	P	N
Colorado *Myotis sp*	P	P	P	P	P	P	P	P	P	P	P	P	N	N	N	P	N	N	P	N	P	N
Mx Oaxaca California human 2008	P	P	P	P	P	P	P	P	P	P	P	P	N	N	N	P	N	N	P	N	P	N

*A comparison of the established reaction patterns of rabies virus (RV) variants£ obtained by indirect fluorescent antibody (IFA) tests with MAbs to the rabies virus nucleoprotein (Rab-N-Mabs). MAb TUC, reaction patterns not shown because data are not available for all RV variants; Tadarida brasiliensis and CA/MX human were TUC positive.

†Associated with resident reservoir host species in North and South America. Rab-N-MAbS: C1-C20 (4); CR54; MAB 54 (5); P41, TUA (TU 187) and TUC (6). Results: P, positive; N, negative; D, diminished reaction; V, varies within the host species reservoir.

‡Varies but most likely positive.
